# Genome-wide identification and expression analysis of expansin gene family in common wheat (*Triticum aestivum* L.)

**DOI:** 10.1186/s12864-019-5455-1

**Published:** 2019-02-01

**Authors:** Zhisheng Han, Yanlin Liu, Xiong Deng, Dongmiao Liu, Yue Liu, Yingkao Hu, Yueming Yan

**Affiliations:** 1 College of Life Science, Capital Normal University, Xisanhuan Beilu No. 105, 100048 Beijing, People’s Republic of China; 2 Hubei Collaborative Innovation Center for Grain Industry (HCICGI), Yangtze University, Jingzhou, 434023 China

**Keywords:** Wheat, Expansin genes, Phylogenetics, Expression, Abiotic stresses

## Abstract

**Background:**

Expansin loosens plant cell walls and involves in cell enlargement and various abiotic stresses. Plant expansin superfamily contains four subfamilies: α-expansin (EXPA), β-expansin (EXPB), expansin-like A (EXLA), and expansin-like B (EXLB). In this work, we performed a comprehensive study on the molecular characterization, phylogenetic relationship and expression profiling of common wheat (*Triticum aestivum*) expansin gene family using the recently released wheat genome database (IWGSC RefSeq v1.1 with a coverage rate of 94%).

**Results:**

Genome-wide analysis identified 241 expansin genes in the wheat genome, which were grouped into three subfamilies (EXPA, EXPB and EXLA) by phylogenetic tree. Molecular structure analysis showed that wheat expansin gene family showed high evolutionary conservation although some differences were present in different subfamilies. Some key amino acid sites that contribute to functional divergence, positive selection, and coevolution were detected. Evolutionary analysis revealed that wheat expansin gene superfamily underwent strong positive selection. The transcriptome map and qRT-PCR analysis found that wheat expansin genes had tissue/organ expression specificity and preference, and generally highly expressed in the roots. The expression levels of some expansin genes were significantly induced by NaCl and polyethylene glycol stresses, which was consistent with the differential distribution of the *cis*-elements in the promoter region.

**Conclusions:**

Wheat expansin gene family showed high evolutionary conservation and wide range of functional divergence. Different selection constraints may influence the evolution of the three expansin subfamilies. The different expression patterns demonstrated that expansin genes could play important roles in plant growth and abiotic stress responses. This study provides new insights into the structures, evolution and functions of wheat expansin gene family.

**Electronic supplementary material:**

The online version of this article (10.1186/s12864-019-5455-1) contains supplementary material, which is available to authorized users.

## Background

Common wheat (*Triticum aestivum* L.) is one of the three most important grain crops worldwide, accounting for about 35% of the world's staple food. Meanwhile, wheat also serves as an important protein source of human food. Hexaploid wheat contains A, B and D genomes with about 17 Gb in genome size, which was five times larger than that of humans [1]. The growth of plant cells is closely related to cell wall that must not only withstand the internal turgor pressure, but also ensure the extension of the cells during plant growth [2, 3]. Expansins are plant cell-wall loosening proteins that involved in cell enlargement and a variety of other developmental processes as well as various abiotic stresses [4], and thus, expansins play important roles in crop growth and development as well as ultimate yield formation.

Expansins were first found in the study of acid-induced cell wall elongation in cucumber hypocotyls, and then identified, isolated and purified from the hypocotyls of cucumber seedlings [5]. Subsequently, expansin genes were widely investigated in different plants, including oat coleoptiles [6], rice [7], cotton fiber [8], and soybean [9]. Expansin proteins normally contain 250-275 amino acid residues, which consists of two domains: N-terminal domain I with 120 to 135 amino acid residues and C-terminal domain II with 90 to 120 amino acids. A signal peptide of 20-30 amino acid residues is present at the N-terminus. Domain I, rich in cysteine, is considered as an important catalytic domain. This region shares some homology with the catalytic domain of the β-1,4-D-endo-glucanase from *Trichoderma* [10]. However, subsequent study showed that β-1,4-D-endo-glucanase cannot induce cell wall elongation although it has obvious catalytic activity, thus the expansin family does not have the activity of hydrolyzing β-glucon sugar [11]. Domain II is speculated to be a polysaccharide binding domain with about 50% similarity to Group-II pollen allergen protein (G2A family) [12], and contains a series of conserved tryptophans [13]. The G2A family proteins consist of two superimposed β-sheets, similar to immunoglobulin. When pollens are secreted onto the stigma, the pollen allergen relaxes the cell wall, so expansins may have similar function. In addition to the G2A family, no other proteins containing domain II homologues are found [10].

According to the nomenclature of Kende et al. [14], plant expansin superfamily is divided into four subfamilies: α-expansin (EXPA), β-expansin (EXPB), expansin-like A (EXLA) and expansin-like B (EXLB). The α-expansin is mainly found in both dicotyledonous plants and monocotyledonous plants of non-*Poaceae*, while β-expansin is predominantly present in monocotyledonous plants. Although EXLA and EXLB have two typical domains of expansin proteins, few experimental reports showed the cell relaxation activity of these two family members [11]. Studies showed that the α- and β-expansin gene subfamilies already existed before the disorganization of vascular plants and bryophytes, and the recent ancestral era of expansin-like A and expansin-like B subfamily can be traced back to gymnosperms and angiosperms [15, 16].

Stretching and relaxation of plant cell walls are achieved by a process of the slow microfibril creep, in which parallel microfibrils and their assemble sugars are separated from each other [17]. The pressure of cell wall provides energy to overcome the viscous resistance of intertwining between cell wall multimers. In living plants, the pressure of cell wall comes from intracellular turgor pressure. This molecular motility occurs only when the relaxation of the cell wall is caused by expansin or some other factors. Otherwise, the cellulose fibrils on the cell wall are tightly tangled with the stromal polysaccharide in situ [18].

The expression of plant expansin genes has obvious tissue specificity [7, 19]. Functional studies have shown that expansins are involved in many developmental processes such as plant growth [20], root hair growth [9, 21], leaf growth [22], fruit softening and ripening [23, 24], seed size and germination [25], pollen tube growth [26, 27] and salinity stress response [28]. In wheat, Lin et al. isolated 18 wheat expansin genes and found that the expression of expansin gene is closely related to the growth and development process [29]; Zhang et al. identified 128 wheat expansin genes using the previous wheat genome database (TGACv1), and found that some of them participated in cold stress response [4]. Carolina et al. found that expansins expression is associated with grain size dynamics in wheat [30]. The over-expression of *TaEXPA2* gene in tobacco improved salt stress tolerance [31], drought stress tolerance [32] and Cd stress tolerance [33]. The over-expression of *TaEXPA2* gene in *Arabidopsis* plants improved oxidative stress tolerance [34]. The over-expression of *TaEXPB23* gene in tobacco improved both oxidative stress tolerance [35] and salt stress tolerance [36]. Expansins may involve in increasing phosphorus availability by regulating the growth and development of plant roots [37]; Expansin also plays important roles in dealing with drought stresses in wheat [38–41]. However, in-depth studies on the structural features, molecular evolution and functional properties of wheat expansin gene family are still needed.

This work aims to carry out a comprehensive study on the molecular characterization, phylogenetic relationship and expression profiling of wheat expansin gene family using the recently released wheat genome database (IWGSC RefSeq v1.1 with a coverage rate of 94%). Our results provide new evidence for further understanding the structure, evolution and function of plant expansin genes.

## Results

### Genome-wide identification and phylogenetic relationship of wheat expansin genes

**Fig. 1:**
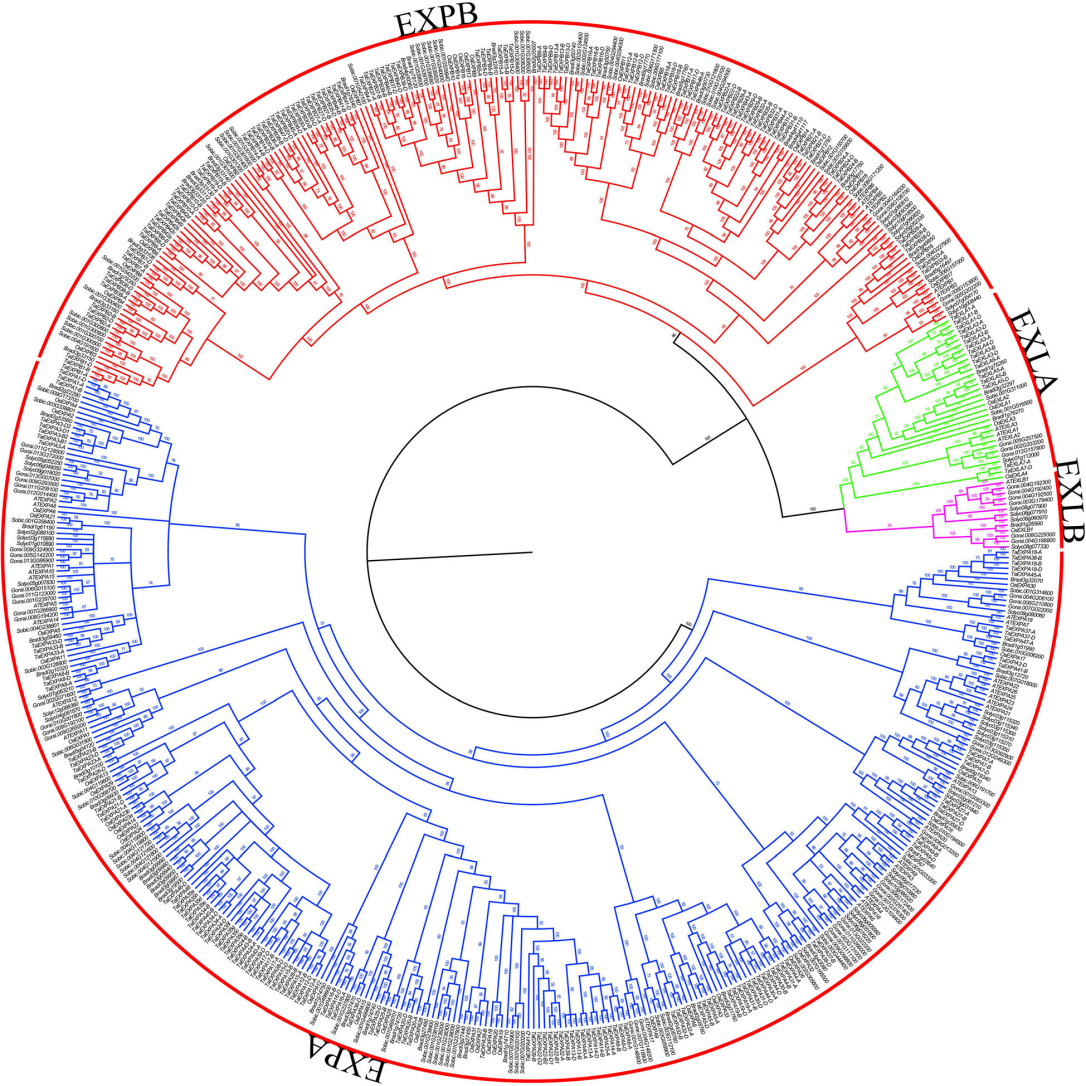
Bayesian phylogenetic tree of all of the expansin proteins from *Triticum aestivum*, *Oryza sativa*, *Brachypodium distachyon* and *Sorghum bicolour Arabidopsis thaliana*, *Solanum lycopersicum* and *Gossypium raimondii*. Clade of blue branches refers to the EXPA subfamily; clade of red branches refers to the EXPB subfamily; clade of green branches refers to the EXLA subfamily; clade of pink branches refers to the EXLB subfamily

Through blast search against the *Triticum aestivum* genome database from GRAMENE (http://ensembl.gramene.org/), a total of 241 wheat expansin genes were obtained. To obtain more information of the expansin superfamily, genome-wide identification of the expansin genes from *Brachypodium distachyon*, *Sorghum bicolor*, *Solanum lycopersicum* and *Gossypium raimondii* genome database was performed. Based on the multiple alignments of the full-length sequences of expansins, two softwares MEGA 5.0 and MrBayes 3.2 were used to construct the phylogenetic trees, including neighbor-joining (NJ) phylogenetic tree (Additional file 1: Figure S1) and Bayesian phylogenetic tree (Fig. 1).

Comparative analysis showed that the topological structures of the two phylogenic trees were generally similar, with only minor differences. Bayesian tree constructed had more advantages in accuracy than the other tree-building methods, which was considered as more credible results for subsequent analysis [42, 43]. The seven plant species were used in this study, including four monocotyledonous plants (*Triticum aestivum*, *Oryza sativa*, *Brachypodium distachyun* and *Sorghum bicolor*) and three dicotyledonous plants (*Arabidopsis thaliana*, *Solanum lycopersicum* and *Gossypium raimondii*). The results from Bayesian tree showed that the four subfamilies (EXPA, EXPB, EXLA and EXLB) of plant expansin genes were classified while wheat expansin genes had no EXLB subfamily (Fig. 1). The size of each subfamily in seven plant species shown in Additional file 2: Table S1 demonstrated that the distribution of expansin genes in the four subfamilies was quite uneven and EXPA was the largest subfamily. EXLA and EXLB were the least-abundant subfamily, only one member of the EXLB family was present in *Arabidopsis* and *Oryza sativa*. In addition, the EXPB family members in the monocotyledonous *Poaceae* were much greater than those in the other three dicotyledonous plants (Fig. 1).

### Molecular characterization of wheat expansin genes

All wheat expansin genes identified were named according to the standard principles for consistency [14], and their information were listed in Additional file 3: Table S2. Among them, *TaEXPA1-29*, *TaEXPB1-24* and *TaEXLA1-4* were named previously [4, 34, 44–46], so the newly identified expansins were named as *TaEXPA30-48*, *TaEXPB27-49*, and *TaEXLA5-7*. The isoelectric points of the expansin family members ranged from 4.64 to 9.78 with an average of 7.95, showing a weakly alkaline. The average molecular weight was 28427.76 Da, ranging from 16305.76 to 39105.36 Da. The number of amino acid residues varied between 150 and 372, and the signal peptide of expansin family members ranged from 10 to 68 amino acids in length. The number of the predicted DPBB_1 domains was 37–91 amino acids in *TaEXPAs*, 72–92 in *TaEXPBs* and 71–80 in *TaEXLAs*. The number of the predicted Pollen_allerg_1 domains was 32–79 amino acids in *TaEXPAs*, 56–93 in *TaEXPBs* and 83–100 in *TaEXLAs*. In general, the expansin members from same subfamily showed similar properties, but those from different subfamilies had significant differences. These results indicate that plant expansins may adapt to different functional requirements by changing the length of its amino acids and physical/ chemical properties.

**Fig. 2:**
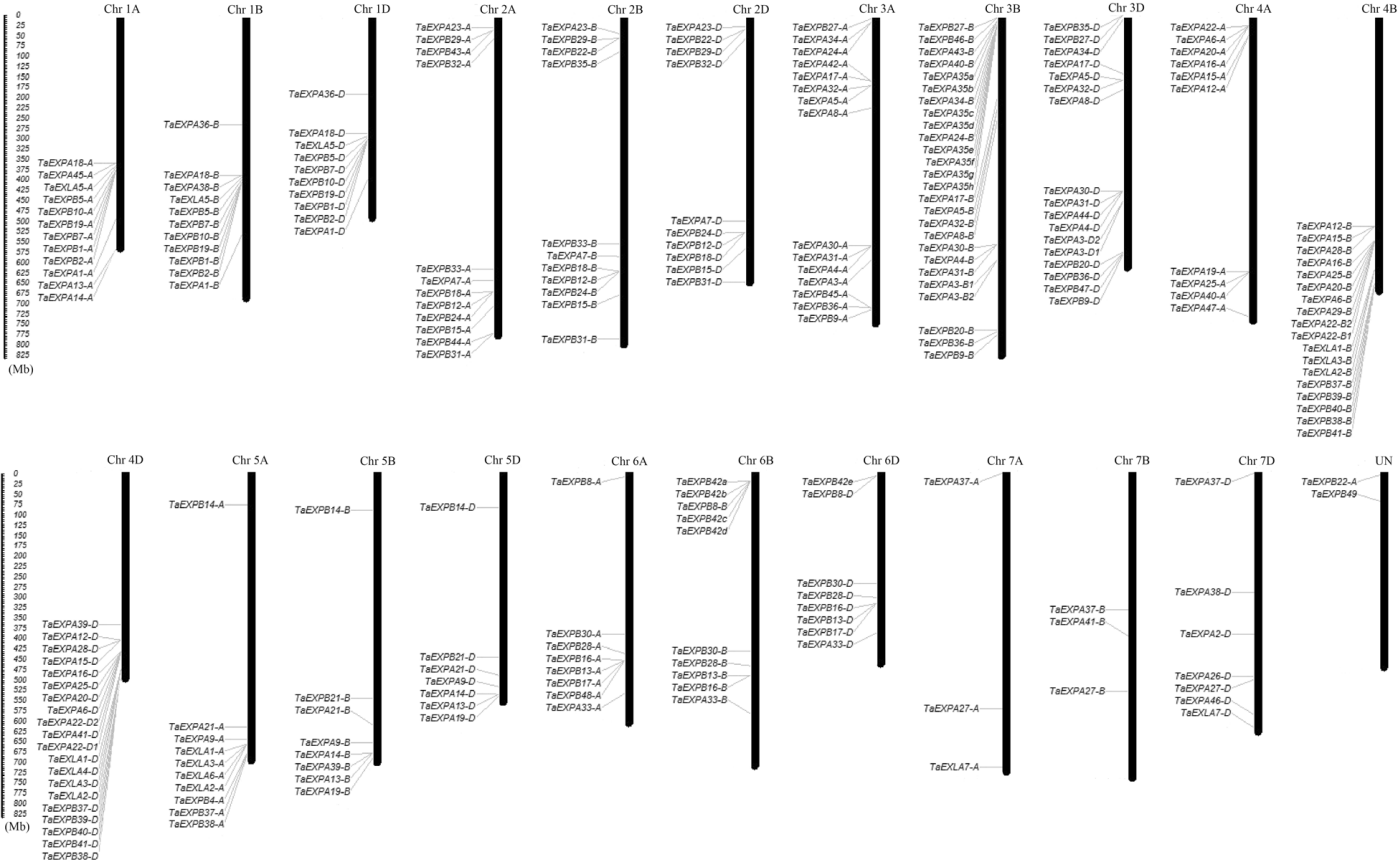
Chromosomal distribution of wheat expansin genes. The figure was produced using the Map Inspector program

Based on *Triticum aestivum* (IWGSC) genome database, the physical positions of the expansin genes to corresponding chromosomes were shown in Fig. 2. All expansin genes identified could be mapped on the chromosomes from 1A to 7D. Obviously, the distribution of expansin genes on the different chromosomes was uneven. Particularly, the chromosome 3B with 26 expansin members had the highest density, but the chromosomes 7A and 7B contained no more than three expansin genes. Almost all of the expansin protein genes had three copies from chromosomes A, B and D such as *TaEXPA1*, *TaEXPA4*, *TaEXPA5*, *TaEXPB1*, *TaEXPB2*, *TaEXPB5*, *TaEXLA1*, *TaEXLA2* and *TaEXLA3*.

**Fig. 3:**
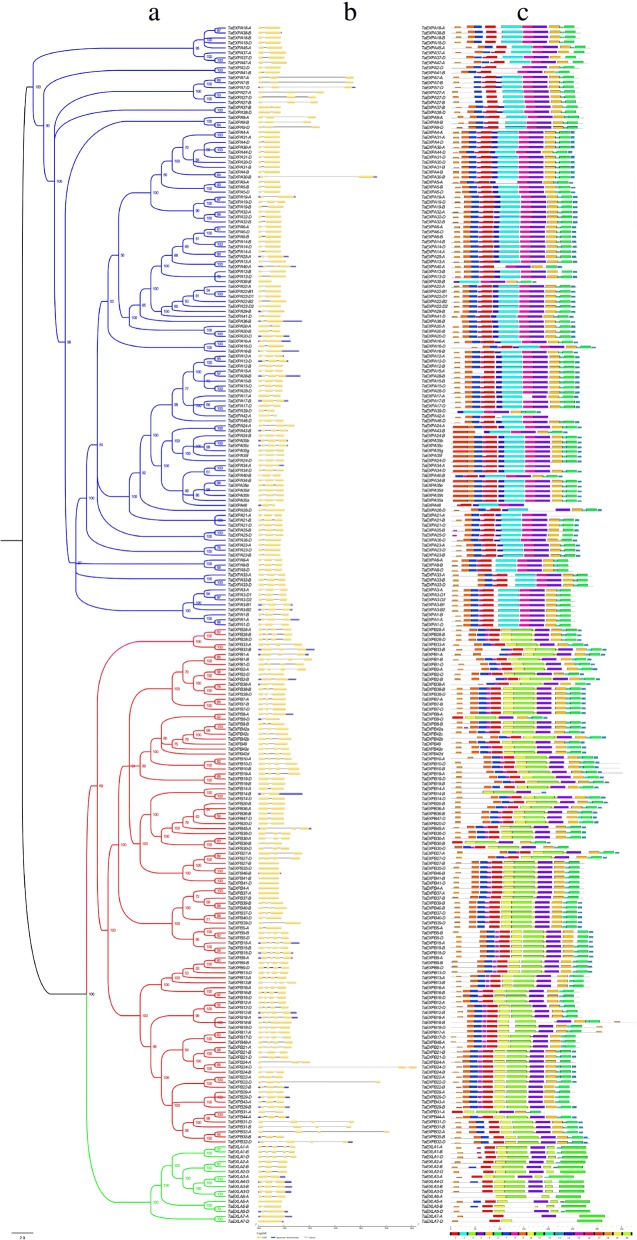
Phylogenetic relationships, exon-intron and motif structures of wheat expansin genes. **a** The rooted Bayesian phylogenetic tree was reconstructed based on a complete protein sequence alignment of 241 expansin genes identified using MrBayes 3.2. Subclades colors indicate the three corresponding gene subfamilies. **b** Exon-intron structures of the expansin genes. Boxes: exons; lines: introns. The lengths of boxes and lines are scaled based on gene length. **c** MEME motif search results. Conserved motifs are indicated in numbered, colord boxes

A sequence alignment of wheat expansin proteins was performed, and a Bayesian phylogenetic tree was constructed (Fig. 3a), the intron-exon distribution of the expansin genes in wheat was analyzed by comparing the CDS sequences and the complete gene sequences. The results showed that the members in the same subfamily had similar structures, and most of them had the same number of exons (Fig. 3b). For example, most members of the EXPB subfamily had four exons, and most members in the EXPA subfamily had three exons and the others generally had two or four exons. These differences may be resulted from the absence or gain of exons during long-term evolutionary processes.

Twenty different motifs were detected in wheat expansins (Fig. 3c), and their structural features were showed in Additional file 4: Figure S2. In the same subfamily, the order, type and number of motifs in expansins were similar, but divergence occurred between different subfamilies. In EXPA subfamily, more than 90% (109/121) of the members had motif 1, 2, 4, 5, 6, 7, 8, 9, 12 and 16 with the same order. This is in marked contrast to the other two subfamilies in which motifs 2, 8, 14 and 17 were absent. The other subfamilies contained motifs 3 and 10, and almost all of the EXPA and EXPB subfamilies had the motifs 1, 4, 5, 6, 7, 9, 12 and 18. These results suggest that EXPA and EXPB subfamilies have a more recent evolution and close phylogenetic relationships. The motif distribution of the entire family members showed that all the subfamilies were highly conservative, but some divergences among different subfamilies still occurred. The high similarity between the sequences and intron-exon structures suggest that wheat expansin genes may have undergone gene duplication events during evolution process.

### Functional divergence analysis of wheat expansins

In this study, type-I and type-II functional divergences were estimated based on Bayesian phylogenetic tree constructed from 241 members of wheat expansin gene family, and the results were showed in Table 1. Type-I functional divergence refers to the evolution of amino acid sites in the evolutionary process occurred after the evolution of the rate of change while type-II functional divergence refers to changes in the physical and chemical properties of amino acid sites [47, 48]. The coefficients of Type-I functional divergence between subfamily pairs EXPA/EXPB, EXPA/EXLA and EXPB/EXLA were statistically significant (θ > 0, likelihood ratio test statistic > 4.96; *p* < 0.01), indicating that significantly different site-specific shifts in evolutionary rate may have taken place at certain amino acid sites between these pairs. Critical amino acid sites were identified in three groups of expansin subfamilies for the analysis of Type-I functional divergence. In order to reduce the occurrence of false positives, Qk > 0.8 is taken as the threshold of important amino acid sites, and the site of Qk < 0.8 is not considered. As shown in Table 1, six type-I functional divergence amino acid sites were detected between EXPA and EXLA subfamilies: 131E, 139Q, 144I, 146P, 161R and 164I, while subfamily pairs EXPA/EXLA and EXPB/EXLA had 45 and 46 sites, respectively (amino acid reference sequence from TaEXPA1-A). These data indicate that a significant change in the rate of evolution may have occurred at these amino acid sites. Besides, compared with EXPA/EXPB, EXPA/EXLA and EXPB/EXLA had relatively larger coefficients of functional divergence (θI) and more sites that were related to functional divergence. Therefore, the functional divergence that exists in EXPA/EXLA and EXPB/EXLA is more significant compared with that present in EXPA/EXPB. No type-II functional divergence sites were identified in this study, because the coefficients of type-II functional divergence between subfamily pairs EXPA/EXPB, EXPA/EXLA and EXPB/EXLA were not statistically significant (θII < 0).

**Table 1:** Functional divergence between subfamilies of the expansins

Group I	Group II	Type-I θI ± s.e.	LRT	Qk> 0.80	Critical amino acid sites
EXPA	EXPB	0.510± 0.074	41.57095	6	131E,139Q,144I,146P,161R, 164I
EXPA	EXlA	1.045± 0.153	42.87446	46	83E,84L,85R,126H,127F,128D,129M,130A,131E,132P,133A,134F,135L,136H,137I,138A,139Q,141R,143G,144I,145V,146P,147V,148S,152F,156K,157K,158G,160I,161R,163T,164I,165N,166G*,168S,169Y*,170F*,171N,182G*,183D,184A,185Q,186S,187V,188S,189I
EXPB	EXlA	1.152±0.148	45.60237	45	83E,84L,85R,126H,127F,128D,129M,130A,131E,132P,133A,134F,135L,136H,137I,138A,139Q,141R143G,144I,145V,146P,147V,148S,152F,156K,157K,158G,160I,161R,163T,164I,165N,166G*,168S,169Y*,170F*,171N,182G*,183D,184A,185Q,186S,187V,188S,189I

*LRT* Likelihood Ratio Statistic, *Qk* posterior probability; *Sites also responsible for the positive selection

Note: All sites are located on the reference sequence TaEXPA1-A based on the multiple alignment results

### Positive selection and coevolution analysis of wheat expansin superfamily

To detect whether expansin gene family has evolved adaptively, we used the CODEML program in PAML v4.4 package [49] and selected the site models and branch-site models to perform positive selection analysis of wheat expansin superfamily. In the site models, no positive selection sites were identified (Table 2). In the branching site model, only when EXLA was chosen as the foreground branch, four sites (166G, 169Y, 170F and 182G) that had undergone positive selection were detected, and their posterior probability values were greater than 0.85 (Table 3). Positive selection often acts on a small number of sites in a relatively short evolutionary period, so positive selection signal may be annihilated by a broadly-acting negative selection [50]. In contrast, when EXPA or EXPB was selected as the foreground branch, the ω value was low, and no sites with a posterior probability greater than 0.85 were identified. Positive selection test results showed that three subfamilies were under different selection pressure. EXPA and EXPB subfamilies were more conserved relative to the EXLA subfamily. When EXLA was selected as the foreground branch, EXPA and EXPB were selected as background branches, more positive selection sites were identified. Thus, the selection pressure of EXLA subfamily was the greatest among three subfamilies.

**Table 2:** Tests for positive selection among codons of expansin genes using site models

Models	*p* ^*a*^	Estimates of parameters	InL	2⊿l	Positively selected sites^b^
M0	1	ω =0.05125	-8064.27327		None
(one-ratio)					
M3	5	p0 = 0.00000 p1 = 0.66833 p2 = 0.33167	-7934.18358	260.2(M3 vs M0)	None
(discrete)		ω1 = 0.00000 ω2 = 0.02999 ω3 =0.10964			
M7	2	p =1.60629 q = 21.60686	-7907.56503		Not allowed
(beta)					
M8	4	p0 =0.99999 p = 1.60629 q =21.60695	-7907.56552	0.00098(M8 vs M7)	None
(beta&ɯ)		(p1 =0.00001) ω =1.17268			

Note: ^a^Number of parameters in the ω distribution

^b^Positive-selection sites are inferred at posterior probabilities > 95% with those reaching 99% shown in bold

*Sites were also found to be implicated in the functional divergence

Note: All sites are located on the reference sequence TaEXPA1-A based on the multiple alignment results

**Table 3:** Parameters estimation and likelihood ratio tests for the branch-site models

Cluster	Site class	Proportion	Backgroud	Foregroud	Positive Selected Sites^a^
			ɯ	ɯ	
EXPA	0	1	0.05125	0.05125	NONE
	1	0	1	1	
	2a	0	0.05125	1	
	2b	0	1	1	
EXPB	0	0.94523	0.05111	0.05111	NONE
	1	0	1	1	
	2a	0.05477	0.05111	160.397	
	2b	0	1	160.397	
EXLA	0	0.76842	0.05103	0.05103	166G*,169Y*,170F*,182G*
	1	0	1	1	
	2a	0.32973	0.05103	1.52409	
	2b	0	1	1.52409	

^a^Positive-selection site are inferred at poster probabilities > 80%

*Sites were also found to be implicated in the functional divergence

Note: All sites are located on the reference sequence TaEXPA1-A based on the multiple alignment results

Coevolutionary analysis of amino acid residues does not take into account the evolutionary dependence between amino acids and can well complement the Bayesian method's defects [51]. In this study, we detected five groups co-evolution sites (15C, 16L; 19R, 20Q; 24G, 25G; 46M, 47G; 249A, 250Q) with the CAPS software (Additional file 5: Table S3). Each group of co-evolution sites is not only adjacent, but also related in terms of molecular weight and hydrophobicity (*p* < 0.01).

### Three-dimensional structure prediction and critical amino acid sites identification of wheat expansin proteins

**Fig. 4:**
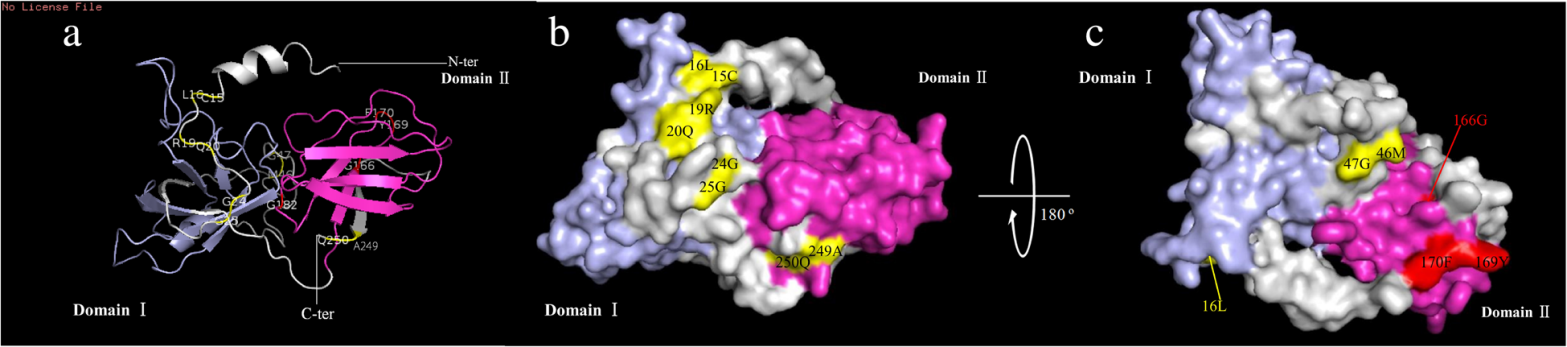
Model building of 3D structure of wheat expansin protein TaEXPA1-A. The N-termintal conserved domain I and C-termintal conserved domain II were marked in blue and pink, respectively. Sites responsible for both functional divergence and positive selection are marked as colored red while sites responsible for coevolution are marked as colored yellow. **a** The overall view of critical amino acid sites on the three-dimensional structure. **b** View of critical amino acid sites on the surface of TaEXPA1-A. Four amino acid sites responsible for both functional divergence and positive selection, five groups amino acid sites related to co-evolution are located on the surface of the 3D structure. **c** 3D structure rotated 180° to show different view sides of the expansin

According to our results, four sites (166G, 169Y, 170F and 182G) are critically important for functional divergence, which also underwent strong positive selection. Thus, these sites could play a key role in the evolution of wheat expansin gene family. The 3-D structure of the representative TaEXPA1-A was constructed by Swiss-model using the homology modeling method, and four key amino acid sites were marked on the 3-D structure (Fig. 4). The 166G, 169Y, 170F and 182G were located on the non-return curl of domain II, and all of them were non-polar amino acid (Fig. 4a). Three sites (166G, 169Y, 170F) were located on the surface of the 3-D structure (Fig. 4b-c), implying that these possible catalytic sites can react more easily with the substrate. In addition, four groups of co-evolution sites were located at the N-terminus of the 3D structure while 249A and 250Q located at the C-terminus of the 3D structure, indicating that these sites may play a key role in maintaining the stability of protein structure and function.

### Analysis of *cis*-acting elements in wheat expansins

The *cis*-acting elements of the promoter region among 241 expansin members were analysed and the results were shown in Additional file 6: Table S4. The results showed that the *cis*-acting elements present in the promoter region of the expansin genes can be divided into seven categories: light responsive elements, development related elements, hormone responsive elements, environmental stress-related elements, promoter related element, site-binding related elements and other elements. Among them, three types of elements related to photoreaction, phytohormone and environmental stress were particularly abundant.

The photoreaction related *cis*-acting elements included G-box, Box 4, GT1-motif and Sp1 etc, of which G-box appeared to be the most abundant, on average, each member contained more than three G-box copies. Phytohormone regulation-related elements were also abundantly present in the expansin promoter region, mainly including ABRE (*cis*-acting element involved in the abscisic acid responsiveness), CGTCA-motifs and TGACG-motifs (*cis*-acting regulatory element involved in the MeJA-responsiveness), and each member contained more than 2.5 copies. The third type of widely distributed *cis*-acting elements was associated with external environmental stress response, of which ARE (*cis*-acting regulatory element essential for the anaerobic induction) and MBS (MYB binding site involved in drought-inducibility) were the most abundant. Other response elements related to environmental stress were also detected, including W-box, GC-motif as well as low temperature induction related LRT and WUN-motif.

### Expression profiling of wheat expansin genes in different organs

The publicly available RNA-seq data generated from bread wheat (var. Chinese Spring) was used to study the expression profiling of 241 wheat expansin genes in different organ**s**, in which 3 genes (*F775_19619*, *F775_14978* and *TRIUR3_08652*) lacked RNA-Seq atlas data because these genes come from *Triticum urartu* (ASM34745v1) and *Aegilops tauschii* (ASM34733v1). The expression data of 238 genes showed that wheat expansin genes had a relatively low transcriptional abundance (Additional file 7: Table S5). Some members expressed only in one organ while some others displayed a wide expression profiling. Specifically, 34.5% (82/238) of expansin genes expressed in all five organs (grain, leaf, root, spike and steam) such as *TaEXPA1-A*, *TaEXPA9-B*, *TaEXPB1-B*, *TaEXLA5-A* and *TaEXLA1-B*. Interestingly, 14.2% members (34/238) specifically expressed in roots such as *TaEXPA13-A*, *TaEXPA14-B* and *TaEXPB15-A*; 27.3% members (65/238) had root expression preference such as *TaEXPA16-A*, *TaEXPA10-B* and *TaEXPB9-A*. In addition, *TaEXPB18-A*, *TaEXPB37-B*, *TaEXPB39-B* and *TaEXPB40-D* had the highest expression level in wheat spike while *TaEXPB27-A*, *TaEXPB27-B* and *TaEXPB27-D* expressed in grains higher than other organs. However, 14 members did not express at the different growth stages of five organs. Our results also showed that most homologous copies had similar expression patterns and widely expressed in five organs with an high expression level in roots such as *TaEXPB7-A*, *TaEXPB7-B* and *TaEXPB7-D*.

We further used the expression data of 238 wheat expansin genes to construct a heat map (Additional file 8: Figure S3a). The results showed that expansin genes showed clear differential expression in the different developmental stages of five organs. In general, high expression occurred at various stages of root development, especially in the seedling roots such as *TaEXPB17-B*, *TaEXPB31-A*, *TaEXPB31-B*, *TaEXPB28-D*, and *TaEXPB16-A*. In the grains, 18 genes had relatively high expression levels at 2 days post-anthesis (dpa), such as *TaEXPB20-D*, *TaEXPB20-B* and *TaEXLA2-A*. Some of them were also highly expressed at 14 dpa during grain development such as *TaEXPB17-A*, *TaEXLA3-B* and *TaEXLA4-D*. In the leaves, 15 genes showed high expression during leaf development (2 dpa) such as *TaEXPB4-A TaEXLA5-A* and *TaEXPA28-D*, while some genes were highly expressed in the leaves of tillering stage such as *TaEXPB1-B*, *TaEXPB1-D* and *TaEXPB16-D*. And in the spike, 16 genes were only highly expressed at the anthesis stage such as *TaEXPB5-B*, *TaEXPB24-D* and *TaEXPB4-A*. Some genes were highly expressed in the spike at both flag leaf and two nodes detectable stages such as *TaEXPA7-A*, *TaEXPA27-B* and *TaEXPB33-B*. In the stem, some of genes had relatively high expression levels at the early stages of stem elongation such as *TaEXPA9-A*, *TaEXPA9-B*, *TaEXPA9-D*, *TaEXPB1-B* and *TaEXPB1-D*, while *TaEXPB20-D*, *TaEXPB36-A* and *TaEXLA2-A* were only highly expressed in the stem of anthesis stage.

**Fig. 5:**
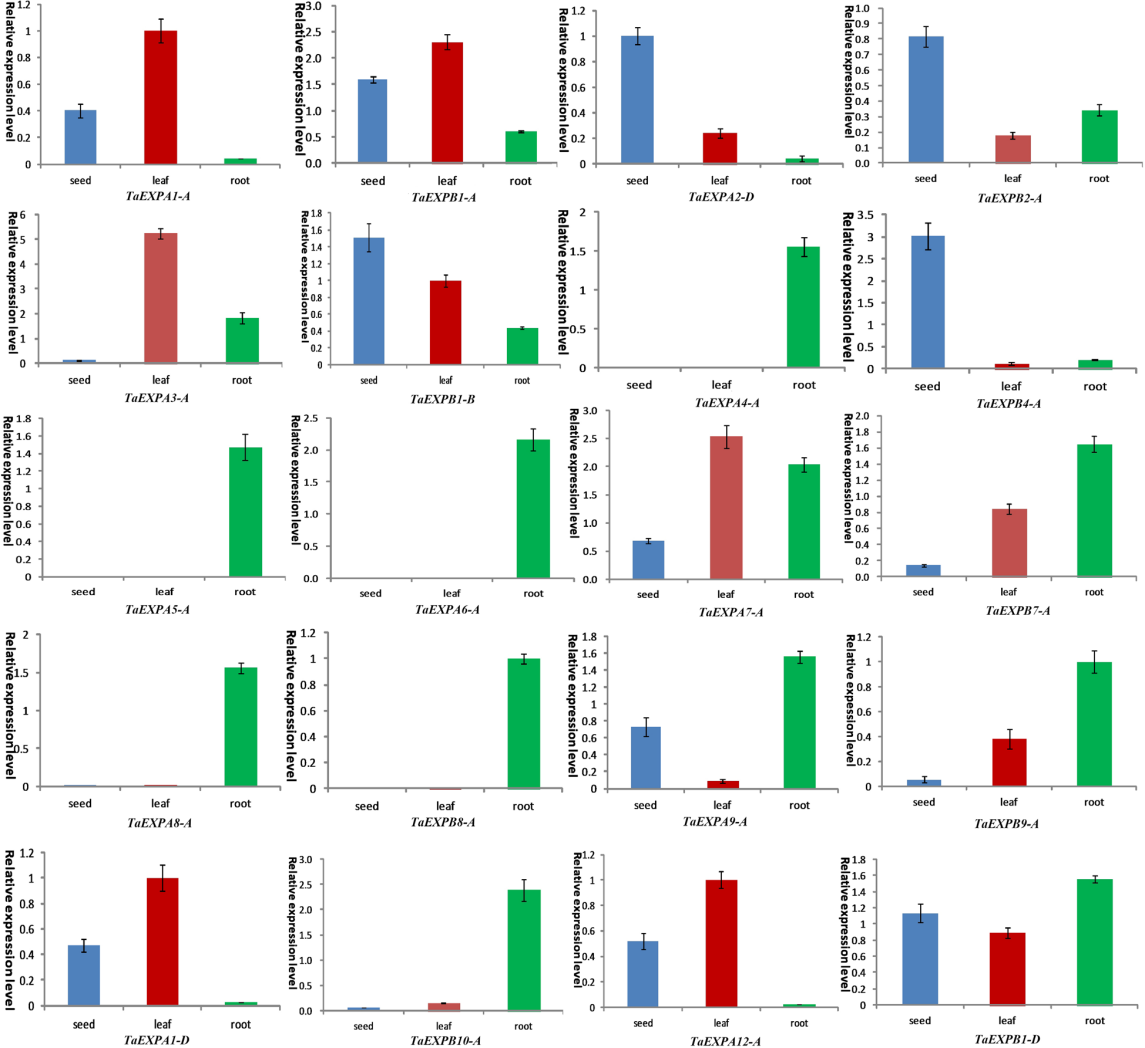
Expression profiling of 20 expansin genes in seed, leaf and root of wheat. Twenty expansin genes included *TaEXPA1-A*, *TaEXB1-A*, *TaEXPA2-D*, *TaEXPB2-A*, *TaEXPA3-A*, *TaEXPB1-B*, *TaEXPA4-A*, *TaEXPB4-A*, *TaEXPA5-A*, *TaEXP6A-A*, *TaEXPA7-A*, *TaEXPB7-A*, *TaEXPA8-A*, *TaEXPB8-A*, *TaEXPA9-A*, *TaEXPB9-A*, *TaEXPA1-D*, *TaEXPB10-A*, *TaEXPA12-A* and *TaEXPB1-D*

To further detect the expression level of wheat expansin genes in different organs (seed, leaf and root), we selected 20 representative genes from EXPA and EXPB subfamily members for quantitative real-time polymerase chain reaction (qRT-PCR) analysis (Fig. 5). The results showed that five genes (*TaEXPA4-A*, *TaEXPA5-A*, *TaEXPA6-A*, *TaEXPA8-A* and *TaEXPB8-A*) specifically expressed in roots while six genes (*TaEXPA7-A*, *TaEXPB7-A*, *TaEXPA9-A*, *TaEXPB9-A*, *TaEXPB10-A* and *TaEXPB1-D*) expressed in all three organs, but had a high expression level in the roots. Four genes (*TaEXPA1-A*, *TaEXPA1-D*, *TaEXPA12-A* and *TaEXPB1-A*) showed the highest expression level in leaves, while *TaEXPA2-D*, *TaEXPB1-B*, *TaEXPB2-A* and *TaEXPB4-A* had a high expression in seeds and low expression in both leaves and roots. In addition, *TaEXPA3-A* highly expressed in leaves, but had a lower expression level in both seeds and roots. These results are generally consistent with the RNA-seq data described above.

### Expression profiling of wheat expansin genes in response to drought and salt stresses

We also used the expression data of 238 wheat expansin genes of seedlings with PEG-simulating drought to construct a heat map (Additional file 8_ Figure S3b). The results showed that 40.3% (96/238) expansin genes were expressed in seedling leaves (9 days after germination) with PEG treatment while 25% (24/96) genes had the highest expression level after 12 h of PEG treatment such as *TaEXPA1-D*, *TaEXPB14-D*, *TaEXPB32-D*, *TaEXPB16-A*, *TaEXPB47-D*, *TaEXPB38-B*, *TaEXPA23-D* and *TaEXPA9-A*. However, 41.7% (40/96) expansin genes were downregulated after PEG treatment such as *TaEXPA3-A*, *TaEXPA9-B*, *TaEXPB1-B*, *TaEXPB7-B*, *TaEXPB10-B*, *TaEXPB2-D* and *TaEXPA27-B*. Particularly, eight genes (*TaEXLA3-D*, *TaEXPA27-D*, *TaEXPB14-B*, *TaEXPB20-B*, *TaEXLA2-D*, *TaEXPB30-B*, *TaEXPB35-B* and *TaEXPB45-A*) showed an upregulation in early stages of seedlings exposed to PEG stress and downregulation after 12 h of PEG treatment.

In order to investigate the expression level of expansin genes in roots and leaves of wheat seedlings with PEG-simulating drought and salt stress, we further selected the same 20 expansin genes shown in Fig. 5 for qRT-PCR analysis. The relative water content (RWC) of leaves showed a significant decrease after drought and salt stress treatments (Additional file 9: Figure S4a-b), and plant phenotypes were also significantly affected by stress treatments, including short seedlings, yellow leaf tip and drooping downwards, and fewer root branches. After 72 hours of stress treatment, the growth of wheat seedlings is severely restricted (Additional file 10: Figure S5).

**Fig. 6:**
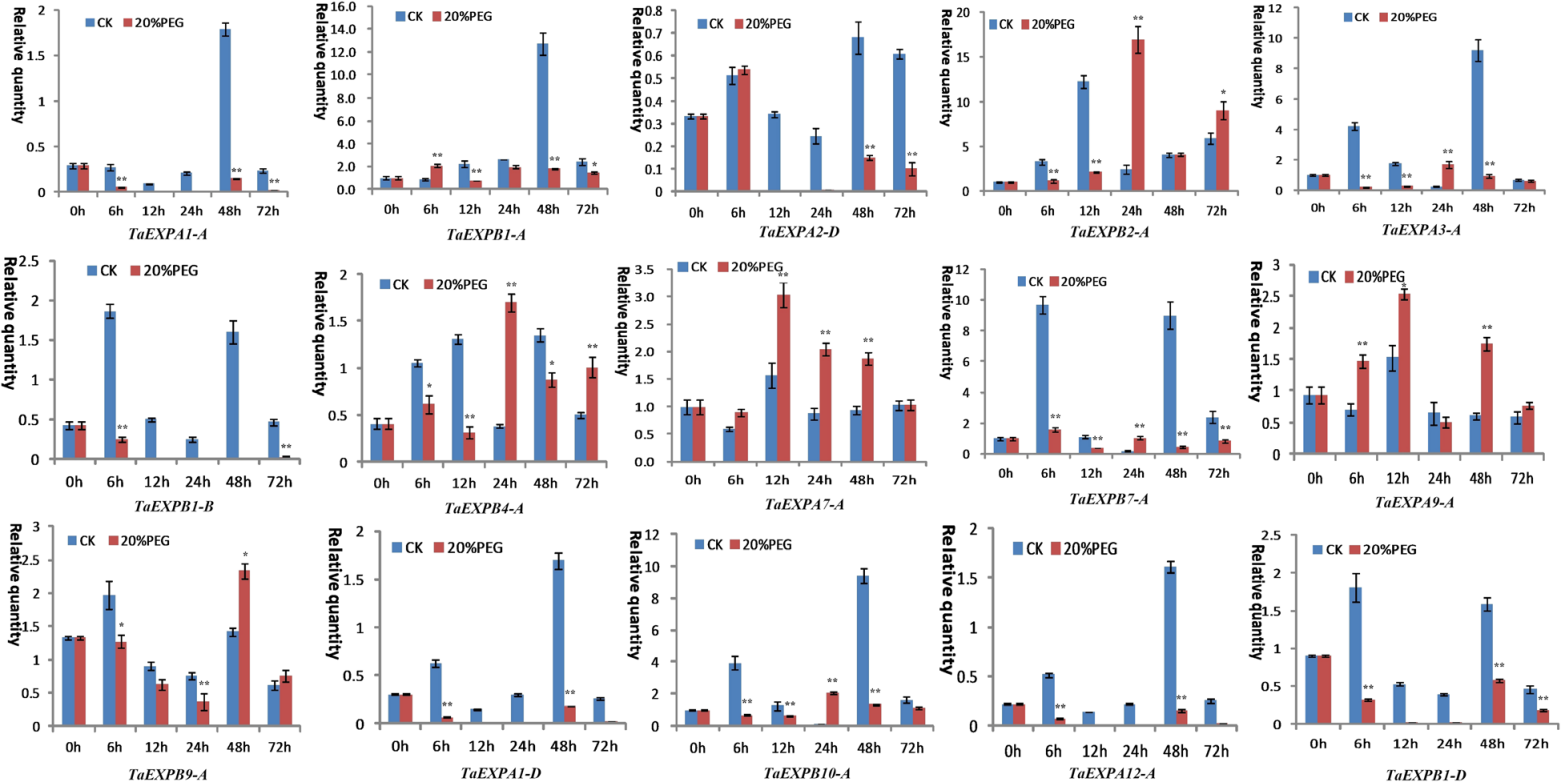
Expression profiling of wheat expansin genes in the leaves under 20% PEG stress. Error bars indicate standard errors of three biological replicates. Statistically significant differences between control group and treatment group were calculated based on an independent Student’s t-tests: * *p* < 0.05; ** *p* < 0.01

**Fig. 7:**
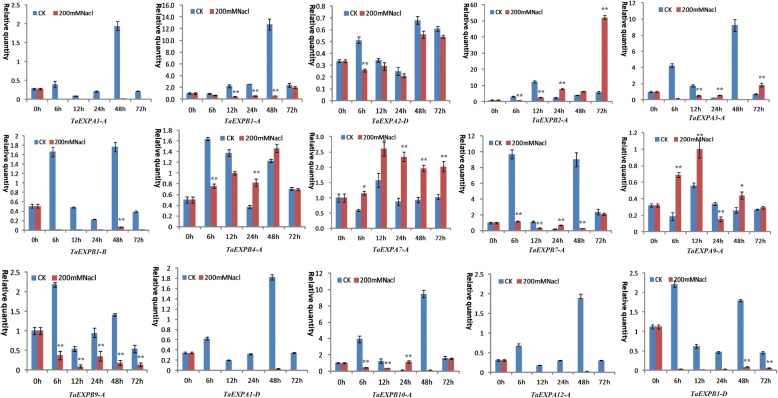
Expression profiling of wheat expansin genes in the leaves under 200 mM NaCl stress. Error bars indicate standard errors of three biological replicates. Statistically significant differences between control group and treatment group were calculated based on an independent Student’s t-tests: * *p* < 0.05; ** *p* < 0.01

qRT-PCR analysis revealed a distinct expression changes of wheat expansin genes in the roots and leaves under drought and salt stresses. In particular, *TaEXPA7-A* in both leaves and roots was upregulated at most of time points after PEG and NaCl stresses. In the leaves, PEG treatment induced an upregulated expression of *TaEXPB2–A* and *TaEXPB4-A* at both 24h and 72h as well as three genes (*TaEXPA3-A*, *TaEXPB7-A* and *TaEXPB10-A*) at 24h. *TaEXPB9-A* displayed an upregulated expression at 48h, and *TaEXPA9-A* was upregulated at all time points under PEG stress (Fig. 6). When subjected to salt stress, the expression of *TaEXPA3-A*, *TaEXPB2-A* and *TaEXPB4-A* increased after 24 hours of the stress treatment, and *TaEXPB10-A* also showed an upregulated expression at 24 h. Particularly, *TaEXPA9-A* was upregulated at all time points of salt stress treatment. The remaining genes generally showed a downregulated expression trend in response to salt stress (Fig. 7).

**Fig. 8:**
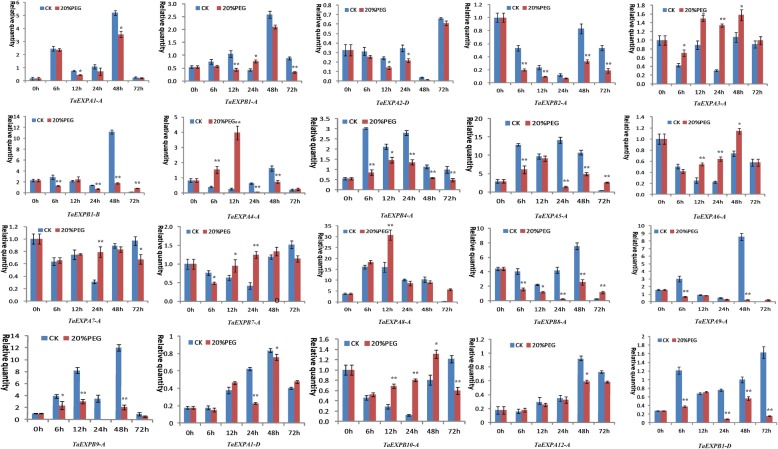
Expression profiling of wheat expansin genes in the roots under 20% PEG stress. Error bars indicate standard errors of three biological replicates. Statistically significant differences between control group and treatment group were calculated based on an independent Student’s t-tests: * *p* < 0.05; ** *p* < 0.01

**Fig. 9:**
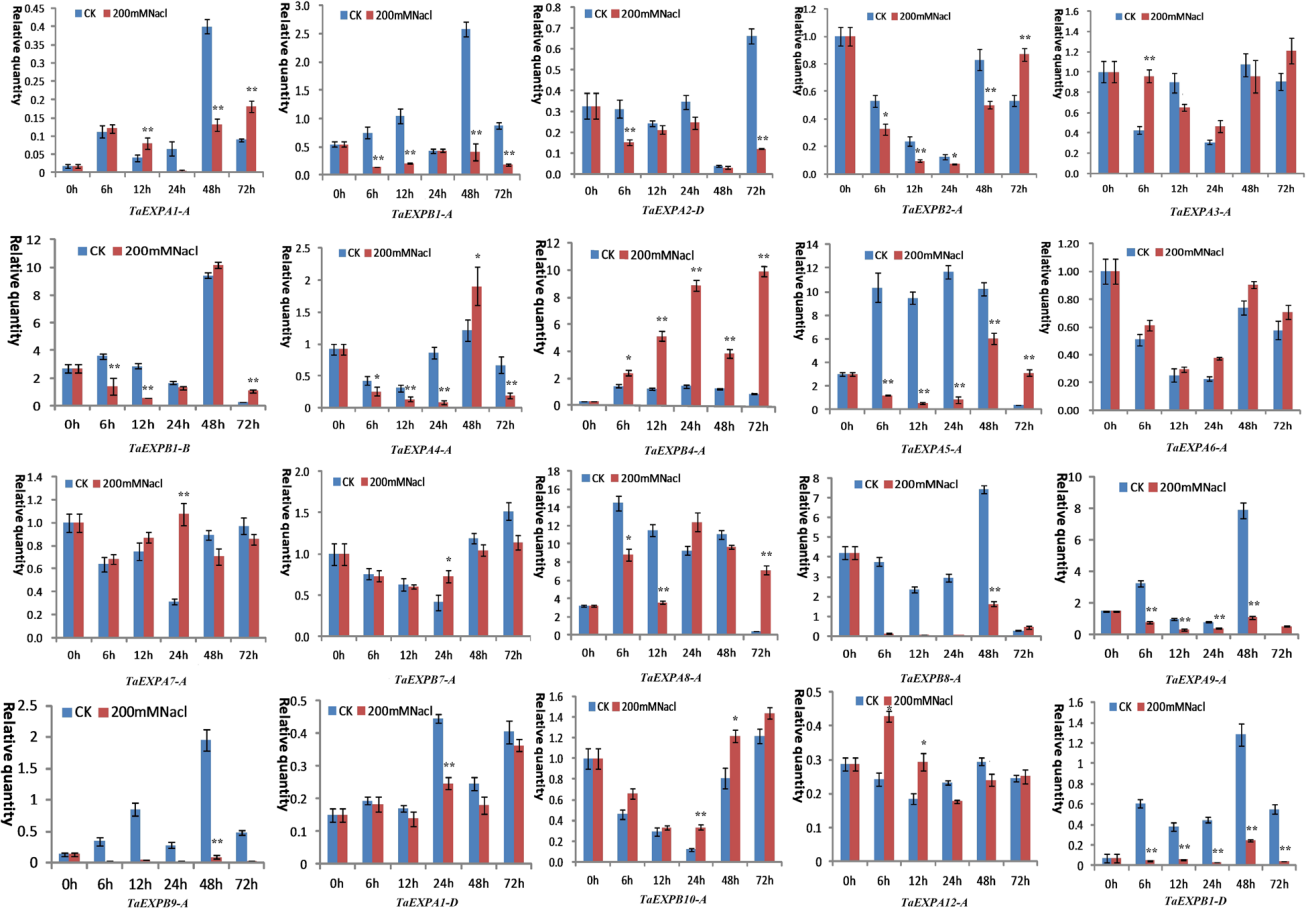
Expression profiling of wheat expansin genes in the roots under 200 mM NaCl stress. Error bars indicate standard errors of three biological replicates. Statistically significant differences between control group and treatment group were calculated based on an independent Student’s t-tests: *p < 0.05; **p < 0.01

In the roots, wheat expansin genes also showed greater expression differences under PEG and salt stresses. Generally, the significant upregulation of wheat expansin genes induced by PEG treatment occurred at different time points such as *TaEXPB10-A*, *TaEXPA3-A*, *TaEXPA4-A* and *TaEXPA8-A* at 6-12 h, *TaEXPB7-A* and *TaEXPA6-A* at 12 h, 24 h and 48 h, and *TaEXPB1-B*, *TaEXPA5-A* and *TaEXPB8-A* at 72 h, *TaEXPA7-A* and *TaEXPB1-A* at 24 h. The other expansin genes were generally downregulated under PEG stress (Fig. 8). When suffered from salt stress, *TaEXPB4-A*, *TaEXPB10-A* and *TaEXPA6-A* showed a significant upregulated expression at all time points of stress treatment. The clear upregulated expression of the other genes occurred at different time points: *TaEXPA12-A*, *TaEXPA7-A* and *TaEXPA3-A* at 6 h, 12 h and 24 h, *TaEXP7-A* and *TaEXPA8-A* at 24 h and 72 h, and *TaEXPA5-A* and *TaEXPB2-A* at 72 h. The remaining genes generally showed a downregulated expression trend (Fig. 9). These results are generally consistent with the RNA-seq data (Additional file 8: Figure S3b), indicated that wheat expansin genes have distinct responses to abiotic stresses due to their functional differentiation. These results are generally consistent with the *cis*-acting elements data described above.

## Discussion

Recent studies have showed that about 70-80% of angiosperms have undergone duplication events [52–54]. Common wheat (*Triticum aestivum* L.) is allohexaploid species with three genomes A, B and D, and more than 85% of sequences are repeats [55]. In this study, the number of wheat expansin genes identified by the recently released wheat genome database was much more than previous reports [4, 34, 45], which is four times of rice. We speculate that polyploidy, tandem duplications, segmental duplications and transposition events are the main mechanisms for the increase of wheat expansin gene copy. On the other hand, our results suggest that the expansin genes in seven plant species have four highly conserved subfamilies (Fig. 1). Interestingly, except for the EXLB subfamily, the other three subfamilies are present in all seven plant species, indicating that these expansins have emerged before the differentiation of these plant species. In particular, the number of EXPB subfamily members in the monocotyledonous is much greater than dicotyledonous plants, consistent with the previous report [56]. In wheat, expansin genes in the same subfamily share similar exon-intron organization and motif composition, but greater structural differences between subfamily are present (Fig. 3), suggesting that expansin genes within subfamily are highly conservative.

Gene duplications are one of the primary driving forces in the evolution of genomes and genetic systems [57]. Divergence of repeated intergenic functions is caused by the accumulation of frequent mutations in amino acid sites [58–60]. By analyzing the function divergence of wheat expansin gene family, we detected 46 sites that play a key role in the type-I functional divergence among subfamily members (Table 1), no type-II functional divergence sites were identified, indicating that a significant change in the rate of evolution may occur at these amino acid sites. Functional divergence and expression differences between repeat genes promote the retention of these genes after a complete genome-wide repeat event [61–63]. At the molecular level, negative selection removes amino acid mutations that impair adaptability, while positive selection retains those amino acid mutations that increase adaptability [64]. The positive selection analysis of wheat expansin gene family detected four sites (166G, 169Y, 170F and 182G) that underwent strong positive selection and led to functional divergence. According to the 3-D structure of the representative TaEXPA1-A, all of these key sites were located on the domain II (Fig. 4), indicating that domain I is more conservative than domain II. In addition, the domain II is the putative polysaccharide binding domain, therefore, these sites may not only exert an important function, but also play a key role in the evolution of wheat expansin gene family.

Protein evolution depends on intramolecular coevolutionary networks, the complexity of which is proportional to the underlying functional and structural interactions among sites [65]. Testing for coevolution between sites is thus an essential step to complement molecular selection analysis and to provide more biologically realistic results. Coevolution analysis of expansin proteins detected five groups of amino acid sites. Interestingly, the amino acid positions of all these groups are located at the N-terminal (4/5) and C-terminal (1/5) signal peptide regions based on the 3-D structure of the representative TaEXPA1-A (Fig. 4). Thus, these sites may play a very important role in the function of the signal peptide to drive the protein into the subcellular organelles of different membrane structures within the cell. These sites may play a key role in maintaining the stability of protein structure and function.

The expression of plant expansin genes is mainly regulated by various hormones, including ABA, auxin, cytokinin, gibberellin, and ethylene. For example, ABA signal transduction may be involved in NaCl-induced accumulation of *TaEXPA2-A* protein in wheat [29, 31]; ethylene affects the expression of *LeEXP1* gene [66]. In this study, we analyzed the *cis*-acting elements of the promoter region of 241 expansin members, and detected a large number of phytohormone regulation-related elements such as ABRE, CGTCA-motifs and TGACG-motifs, each member contains more than 2.5 copies of these elements. The expression of plant expansin genes is also regulated by the development process, and has tissue/organ expression specificity and preference. For example, *GmEXP1* in soybeans showed a big expression difference in different development stages of roots [9]. RNA-seq data and qRT-PCR analysis of *TaEXPA4-A*, *TaEXPA5-A*, *TaEXPA6-A*, *TaEXPA8-A* and *TaEXPB8-A* were specifically expressed in roots whereas *TaEXPA1-A*, *TaEXPA1-D*, *TaEXPA12-A* and *TaEXPB1-A* had clear expression preference in leaves (Fig. 5). This suggests that expansin genes play important roles in the growth and development of different wheat organs.

Expansin genes are also regulated by various environmental factors. For example, the upregulated expression of wheat expansin genes can be induced by oxidative stress [29], salt stress [31] and drought stress [32]. Water status of plants dominates their response to drought and salt stress [67]. When subjected to drought and salt stresses, plants need to maintain higher relative water content and lower water potential in tissues. It may be a common mechanism for adaptability of plants to drought and salt stresses [68, 69]. On the one hand, stress-induced expression of expansin genes promotes the accumulation of expansin and plant root elongation. The growth of plant roots enhances the ability to absorb water in the stress environments. Elevated levels of NaCl in the environment affect the ion selectivity of the cell membrane, eventually affect the homeostasis of Na^+^/K^+^ in plant tissues [70]. Our results showed that many wheat expansin genes were upregulated under high salt stress, these genes may play an important role in maintaining the balance of Na^+^ and K^+^ inside cells absorption. The high expression of expansin gene increased the content of soluble sugar and proline in plant cells, thereby reduced the internal water potential of the cells, made the cells easier to absorb water and reduced their damage under stress conditions [31]. Meanwhile, in order to protect itself from damage under stress conditions, plants may eliminate reactive oxygen species (ROS) through some antioxidant enzymes [71–73]. The expression of wheat expansin genes may promote the upregulation of certain antioxidant-related genes, and reduced ROS accumulation [29]. In this study, we also detected a large number of environmental stress-related elements in wheat expansin genes such as ARE and MBS, and each member contains more than 1.2 copies of these elements, indicated that the expression of expansin genes was affected by external environmental stress such as hypoxia and drought. Other response elements related to external environmental stress such as W-box and GC-motif, the action element related to low temperature induction LRT and WUN-motif were also found in expansin genes. These stress-related elements could confer expansins a potential function in response to abiotic stresses.

## Conclusions

This study identified 241 expansin genes from recently released wheat genome database, which were classified into EXPA, EXPB and EXLA subfamilies. Molecular characterization showed that wheat expansin gene family showed high evolutionary conservation. The branch site model analysis revealed that there was weak selection pressure between subfamilies of expansins. Functional divergence analysis showed that type I divergence was the main cause of the function changes of wheat expansins. Five groups of coevolutionary sites identified in this study may play an important role in maintaining the stability of expansin structure and function. Analysis of wheat expansin *cis*-acting elements showed that the expression of plant expansin genes is regulated by various hormones and various environmental factors. RNA-seq data and qRT-PCR analysis revealed that wheat expansin genes were differentially expressed in different tissues and organs, and had tissue/organ specificity and expression preference, suggesting that wheat expansins had clear function differentiation. The expression profiling under PEG and salt stresses showed that some wheat expansin genes were significantly upregulated, indicating their important roles in response to drought and salt stresses. Our findings provide new insights into the structure, evolution and function of plant expansin gene family.

## Methods

### Genome-wide identification of expansin genes

Genome-wide identification of the expansin gene family from seven species of monocotyledonous and dicotyledonous plants was performed. Firstly, 35 and 58 expansin sequences from *Arabidopsis thaliana* and *Oryza sativa* were respectively obtained from EXPANSIN CENTRAL (http://www.personal.psu.edu/fsl/ExpCentral/). The sequences of the 128 previously identified TaEXP genes [26] were obtained from TGACv1 database (http://www.gramene.org/), and all of these sequences were used to identify new wheat expansin genes using a blast search against the recently released *Triticum aestivum* genome database (IWGSC RefSeq v1.1) with a coverage rate of 94% from GRAMENE (http://ensembl.gramene.org/). The sequences from *Triticum urartu* (ASM34745v1) and *Aegilops tauschii* (ASM34733v1) were used as a second set of supplementary data [74]. We also used these sequences for BLAST searches in the following species: monocotyledonous plants *Brachypodium distachyon* and *Sorghum bicolour*, and dicotyledonous plants *Solanum lycopersicum* and *Gossypium raimondii* genome database in Phytozome v12.1 (https://phytozome.jgi.doe.gov/pz/portal.html). The online tools Pfam (http://pfam.xfam.org/) and SMART (http://smart.embl-heidelberg.de/) were used to screen expansin proteins. Those with only one of two domains or without a complete open reading frame were removed.

### Exon-intron structure, conserved motif, chemical character, genic physical location on chromosomes and *cis*-acting elements analysis

Genomic sequences, coding sequences, protein sequences and promoter sequences of all wheat expansins identified were downloaded from the Phytozome (https://phytozome.jgi.doe.gov/pz/portal.html) and GRAMENE. All gene IDs were unified into IWGSC gene ID. The exon-intron organization of wheat expansin genes was detected by comparing the coding sequences (CDS) with their corresponding genomic sequences by the online tool GSDS (http://gsds.cbi.pku.edu.cn/) [75]. Conserved motifs other than the canonical domain of expansins were detected by the online tool MEME (http://meme-suite.org/tools/meme). The repeat number was set to 0 or 1, the maximum number of motifs to 20, and the rest of the run parameters to system default [56, 76]. In addition, the signal peptide lengths of wheat expansin-encoding genes were predicted by SignalP 4.1 server (http://www.cbs.dtu.dk/services/SignalP/). The theoretical values of the isoelectric point, relative molecular mass and the grand average of hydrophobicity (GRAVY) of wheat expansins were predicted by the Protparam tool (http://web.expasy.org/ protparam/) [77–79]. Map Inspect software (http://mapinspect.software.informer.com/) was used to analyze the genic physical location on chromosomes. *Cis*-acting elements analysis in the 1500 bp upstream regions was performed with PlantCARE (http://bioinformatics.psb.ugent.be/webtools/plantcare/html/).

### Phylogenetic tree construction

Multiple sequence alignments of the identified expansin full-length protein sequences were performed using the MUSCLE software [80, 81]. Based on the alignment files, two softwares MEGA5.0 [82] and MrBayes3.2 [83] were used to construct the phylogenetic tree. When using MEGA5.0, the bootstrap of the phylogenetic tree was set to 1000, and the rest of the parameters were default. Neighbor-joining (NJ) phylogenetic tree was constructed by using adjacency method [84].

### Functional divergence, positive selection and coevolution analysis

The software DIVERGE 3.0 can detect whether there is a significant change in evolution rate at a particular site using maximum likelihood (Type-I functional divergence) or a significant change in the physicochemical properties of the amino acid on the homologous sequence (Type-II functional divergence) [59]. Both Type-I and Type-II functional divergence coefficients (θI, θII) between subfamilies were calculated by DIVERGE3.0. If these coefficients are significantly greater than 0, it implies that the selection constraints or the physicochemical properties of the particular amino acid residues were changed significantly between the two subfamilies after gene duplication or differentiation. In addition, the posterior probability (Qk) predicts the reliability of functional divergence amino acid sites. The higher the Qk value, the higher the probability of functional divergence of type I or type II between two subfamilies. In this study, the critical value of Qk is set to 0.8 [85].

The positive selection analysis for wheat expansin genes was performed using the site models and the branch site models in the Codeml program in PAML4.4 software package [86]. The nucleotide sequence and corresponding protein sequence alignment file were submitted to PAL2NAL [87], the Bayesian tree and multiple sequence alignment files were submitted to the PAML software to calculate the ratio of dN (non-synonymous substitution rate) to dS (synonymous substitution rate) at each site. The magnitude of the value of dN/dS (ω) represents the types of selection: ω < 1 for negative selection, ω = 1 for neutral selection and ω > 1 for positive selection [88]. In the site models, the likelihood ratio test (LRT) was used to test positive selection by comparing the two pairs of models (M0/M3 and M7/M8). M0 is called one-ratio model, meaning that all expansin codons evolve at a single evolutionary rate. M3 is a discrete model that allows each site to evolve at a different rate and calculates the probability of each site having a purification selection (p0), a neutral selection (p1), and a positive selection (p2). The corresponding ω values (ω1, ω2, ω3) can also be derived from the data. The Beta model (M7) is a null test for positive selection, assuming a Beta distribution with ω between 0 and 1. Finally, the Beta & ω model (M8) add one extra class with the same ratio ω1 [89]. The test made by the comparison between M7 and M8 is the strictest of positive selections. The branching site models used each branch of the phylogenetic tree as a foreground branch, while the other branches serve as a background branch, and the sites in the sequence are classified into four categories. The first type of sites are highly conserved in each branch with smaller values of ω (0 < ω0 < 1); the second type of locus is subject to neutral selection or weaker positive selection (ω1=1 or slightly less than 1). Categories III and IV include background branches that are more conservative or neutral-selected and those under positive selection (ω2 > 1). Before the likelihood ratio test (LRT), we should also compare whether there is a significant difference between Mf model (free ratio model) based on LRT test and M0 model, if there is, abandon M0 model, Mf model is established, indicating that different branches do different rate of evolution. The BEB method was used to calculate posterior probabilities (Qks) of sites with ω2 > 1, sites with a posterior probability of > 0.8 are most likely sites of positive selection [47, 90].

To identify coevolution between amino acid sites, a Coevolution Analysis using Protein Sequences (CAPS) was performed with PERL-based software [91]. CAPS provides a mathematically simple and computationally feasible means of comparing the correlated variance of evolutionary rates at two amino acid sites corrected by time since divergence of the protein sequences to which they belong. Blosumcorrected amino acid distance was used to identify amino acid covariation. The phylogenetic sequence relationships were used to remove phylogenetic and stochastic dependencies between sites.

### Three-dimensional structure prediction

The structure of wheat expansin proteins was modeled by searching SWISS-MODEL database (http://swissmodel.expasy.org/) using the amino acid sequence [92], and Pymol software was applied to visualize the three-dimensional structure of expansin proteins.

### Plant materials and stress treatments

Common wheat (*Triticum aestivum* L., AABBDD, 2n=6x=42) Chinese Spring (CS) was used as material in this study. Seeds with full size were selected and sterilized with 70% alcohol and 10% sodium hypochlorite. Then sterilized seeds were put on the wet sterile filter paper in sterilized Petri dishes and shaded for 48 h under natural conditions. Under the condition of 16/8 h light, 25/20°C temperature and 70% relative humidity, the seedlings (15 days after germination) were cultured to two leaves and then respectively transferred into a nutrient solution containing 20% polyethylene glycol (PEG6000) and 200 mM sodium chloride (NaCl) for stress treatment. Meanwhile, seedlings with normal growth condition were used as control. Roots and leaves from control and treatment were collected at 0, 6, 12, 24, 48 and 72 h, quickly frozen with liquid nitrogen and stored in a -80°C for RNA extraction. In addition, CS seeds were planted in the greenhouse, and the samples from five days after pollination (DAP) were collected for RNA extraction. The relative water content (RWC) of seedling leaves and roots was measured according to Lv et al. [93].

### RNA-seq expression analysis

The publicly available RNA-seq data generated from bread wheat var. Chinese Spring was used to study the expression of newly identified wheat expansin genes. These data were collected from five different wheat organs (grain, leaf, root, spike and steam) during developing seedling, vegetative and reproductive stages. And we also collected dates of seedlings with PEG-simulating drought, RNA-seq data of wheat expansin genes were obtained from expVIP (http://www.wheat-expression.com/) [94]. Cluster analysis of the RNA-seq data was performed by employing the Euclidean distance method over a complete linkage dissimilarity matrix using the Cluster 3.0 and TreeView.

### Total mRNA extraction and qRT-PCR

Total RNA from the roots, leaves and seeds at each time points was isolated using TRIzol Reagent (Invitrogen). Purification of total RNA and cDNA synthesis according to the manufacturer’s instructions with PrimeScript® RT Reagent Kit with gDNA Eraser (TaKaRa, Shiga, Japan). The specific primer sequences for expansin genes were designed using Primer 5.0, shown in Additional file 11: Table S6. Cyclophilin (ADP) was used as the reference gene. The sample mixture for qRT-PCR was performed according to the procedures of the previous report [91], and three biological replicates were used for each sample. All data were analyzed with CFX Manager Software (Bio-Rad). The optimal performance was conducted, in which the correlation coefficient (R2) of 0.994-0.999 and PCR amplification efficiency (E) of 90–110% were controlled. Ct values were averaged. The fold change in the target gene relative to the *Triticum aestivum* L. constitutively expressed expansin gene is determined by: Fold Change =2-Δ (ΔCt) where ΔCt = Ct target - CtSamDC and Δ (ΔCt) = ΔCttreated - ΔCtcontrol, according to the Minimum Information for Publication of Quantitative Real-Time PCR Experiments (MIQE) guidelines.

## Additional files

Additional file 1: **Figure S1.** Neighbor-joining (NJ) phylogenetic tree of all of the expansin proteins from *Triticum aestivum*, *Oryza sativa*, *Brachypodium distachyon* and *Sorghum bicolour*, *Arabidopsis thaliana*, *Solanum lycopersicum* and *Gossypium raimondii*. Clade of blue branches refers to the EXPA subfamily; clade of red branches refers to the EXPB subfamily; clade of green branches refers to the EXLA subfamily; clade of pink branches refers to the EXLB subfamily. (JPG 14757 kb)

Additional file 2: **Table S1.** Distribution of four subfamily members in expansin gene families from different plant species. (XLSX 11 kb)

Additional file 3: **Table S2.** The information of expansin gene superfamily in wheat. (XLSX 31 kb)

Additional file 4: **Figure S2.** Schematic diagram of motifs of wheat expansin proteins. The schematic diagram was derived from MEME. The order of motifs of expansin proteins in the diagram was automatically generated by MEME according to scores. (JPG 8356 kb)

Additional file 5: **Table S3.** Coevolution sites in wheat expansin (All sites are located on the reference sequence TaEXPA1-A based on the multiple alignment results). (XLSX 10 kb)

Additional file 6: **Table S4** *Cis*-element analysis of 2000 bp nucleotide sequences data upstream of the translation initiation codon of expansin genes. (XLSX 141 kb)

Additional file 7: **Table S5.** The RNA-Seq atlas data of the expansin genes. (XLSX 48 kb)

Additional file 8: **Figure S3.** RNA-seq expression analysis of wheat expansin genes. The hierarchical cluster color code: the largest values are displayed as the reddest (hot), the smallest values are displayed as the greenest (cool), and the intermediate values are a lighter color of either red or green. Raw data were normalized by the following equation: reads/kilobase/million. (JPG 6660 kb)

Additional file 9: **Figure S4.** Relative water content changes of wheat leaf and root under PEG and salt stress treatments. (JPG 1208 kb)

Additional file 10: **Figure S5.** The wheat seeding changes under 200 mM NaCl and 20% PEG6000. (JPG 236 kb)

Additional file 11: **Table S6.** Primers used for qRT-PCR of expansins in wheat (XLSX 12 kb)

## Abbreviations

ADP: Cyclophilin
CAPS: Coevolution Analysis using Protein Sequences
CDS: Coding sequences
CS: Chinese Spring
DAP: Days after pollination
dN: Non-synonymous substitution rate
dpa: days post-anthesis
dS: Synonymous substitution rate
G2A: Group-II pollen allergen protein
GRAVY: the grand average of hydrophobicity
LRT: the likelihood ratio test
MIQE: Minimum Information for Publication of Quantitative Real-Time PCR Experiments
NaCl: sodium chloride
NJ: Neighbor-joining
PEG: Polyethylene glycol
Qk: Posterior probabilities
qRT-PCR: quantitative real-time polymerase chain reaction
ROS: Reactive oxygen species
RWC: Relative water content
