# Supplementary figures and images for: Genome-wide identification and expression analysis of expansin gene family in common wheat (Triticum aestivum L.)
Source: BMC Genomics. 2019 Feb 1;20:101. doi: 10.1186/s12864-019-5455-1 (PMC6359794; doi:10.1186/s12864-019-5455-1)

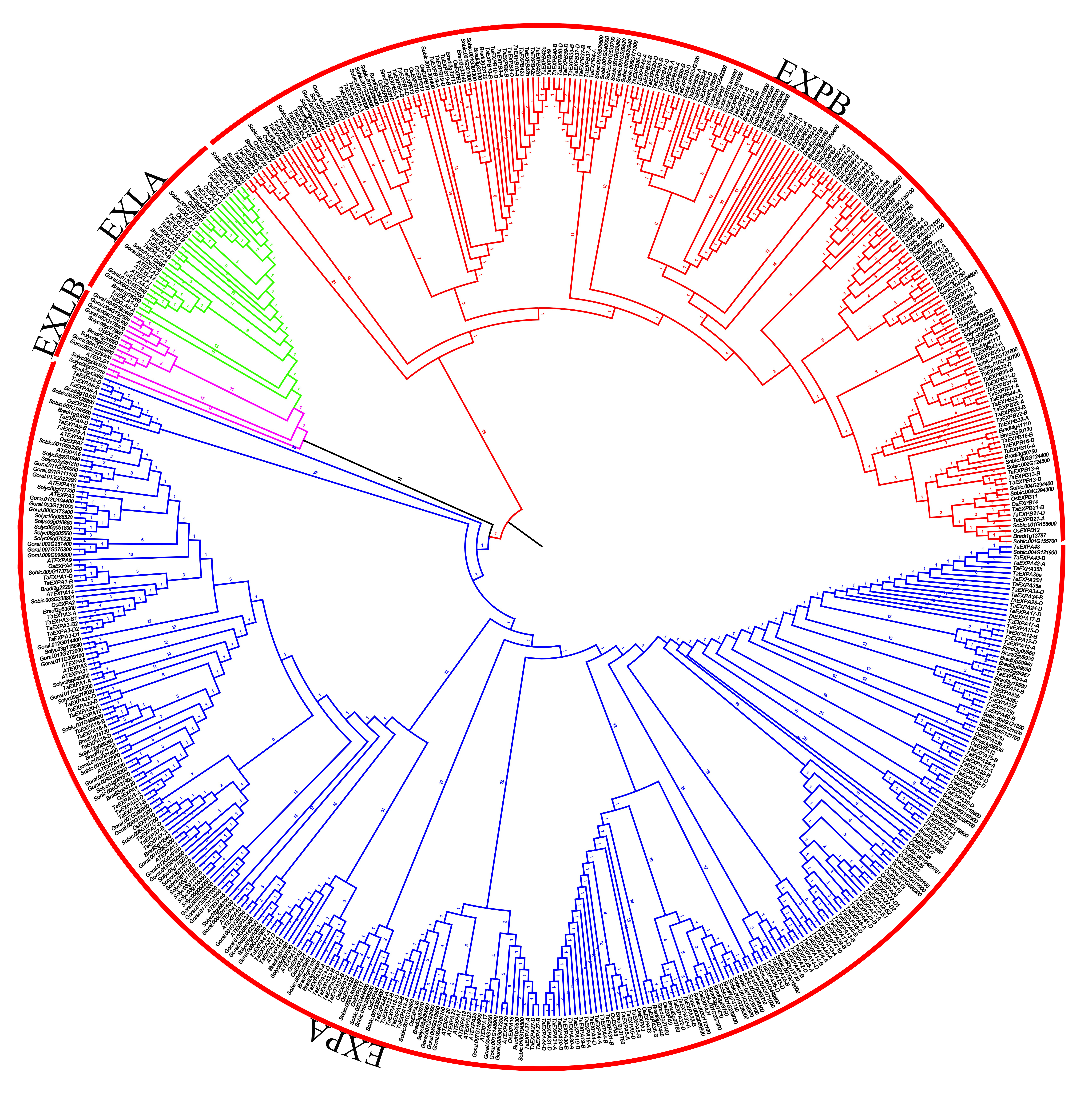

Supplement: Supplementary file 1 — Figure S1. Neighbor-joining (NJ) phylogenetic tree of all of the expansin proteins from Triticum aestivum, Oryza sativa, Brachypodium distachyon and Sorghum bicolour, Arabidopsis thaliana, Solanum lycopersicum and Gossypium raimondii. Clade of blue branches refers to the EXPA subfamily; clade of red branches refers to the EXPB subfamily; clade of green branches refers to the EXLA subfamily; clade of pink branches refers to the EXLB subfamily. (JPG 14757 kb) [file 12864_2019_5455_MOESM1_ESM.jpg]

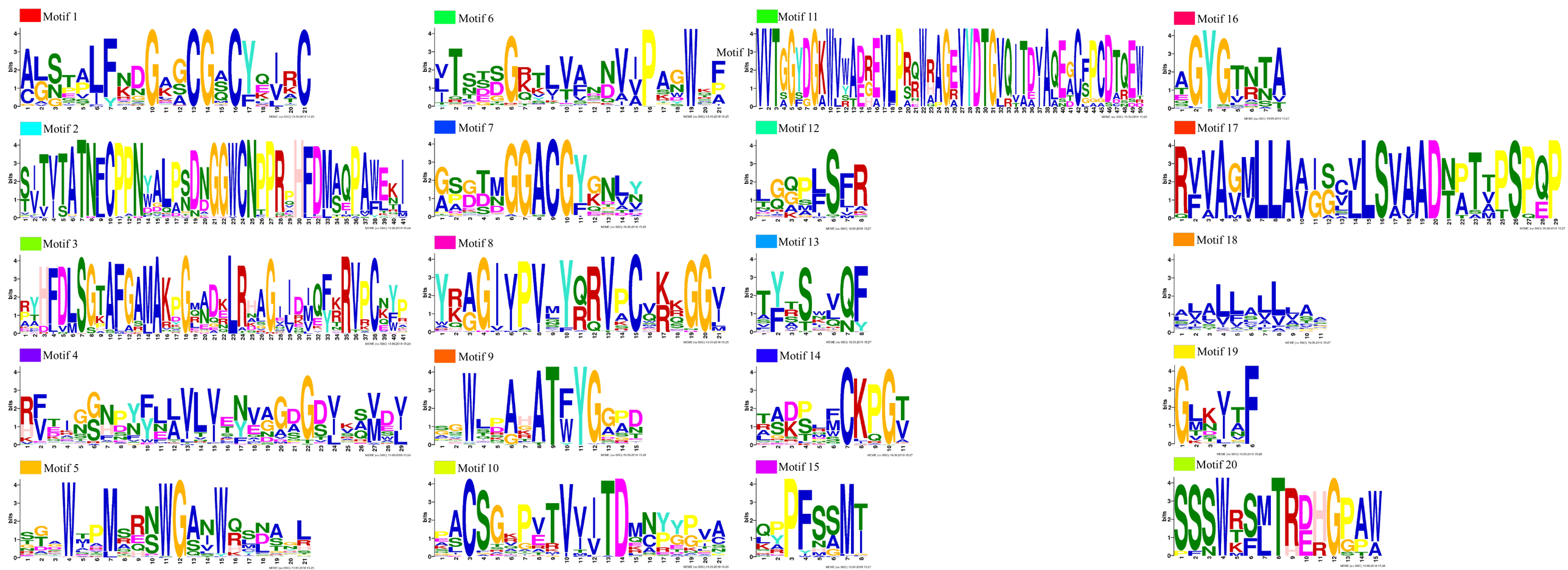

Supplement: Supplementary file 4 — Figure S2. Schematic diagram of motifs of wheat expansin proteins. The schematic diagram was derived from MEME. The order of motifs of expansin proteins in the diagram was automatically generated by MEME according to scores. (JPG 8356 kb) [file 12864_2019_5455_MOESM4_ESM.jpg]

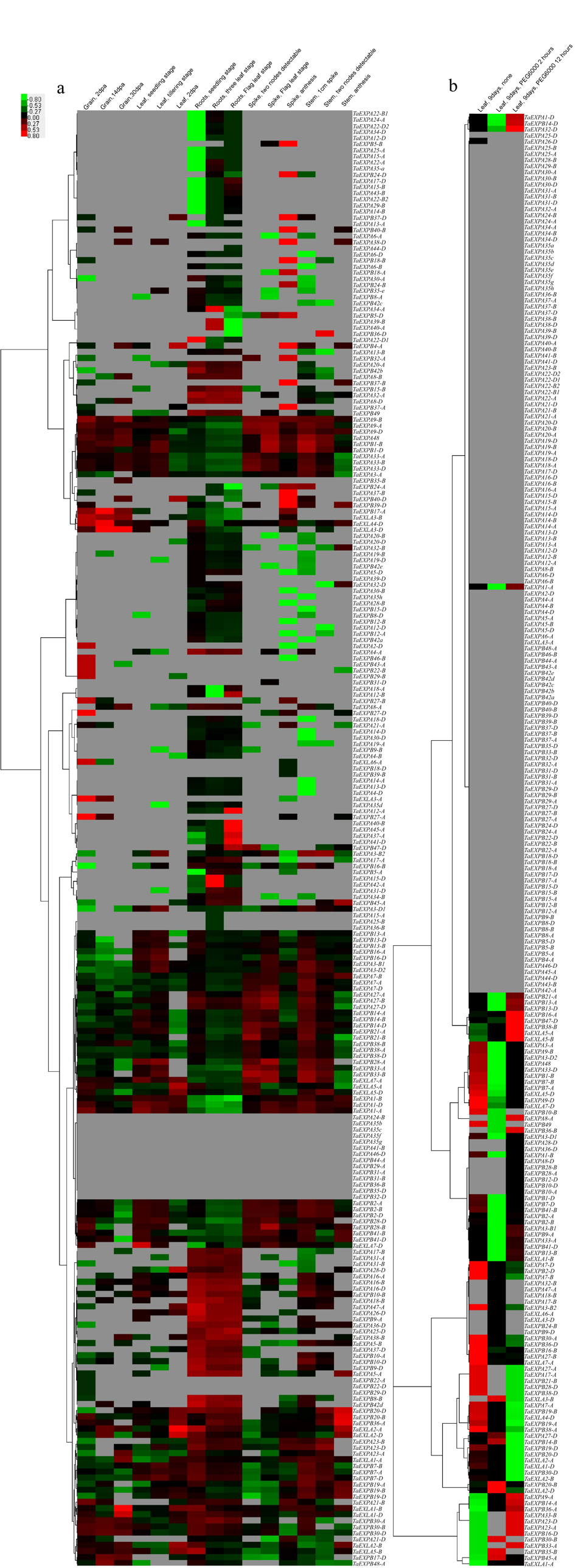

Supplement: Supplementary file 8 — Figure S3. RNA-seq expression analysis of wheat expansin genes. The hierarchical cluster color code: the largest values are displayed as the reddest (hot), the smallest values are displayed as the greenest (cool), and the intermediate values are a lighter color of either red or green. Raw data were normalized by the following equation: reads/kilobase/million. (JPG 6660 kb) [file 12864_2019_5455_MOESM8_ESM.jpg]

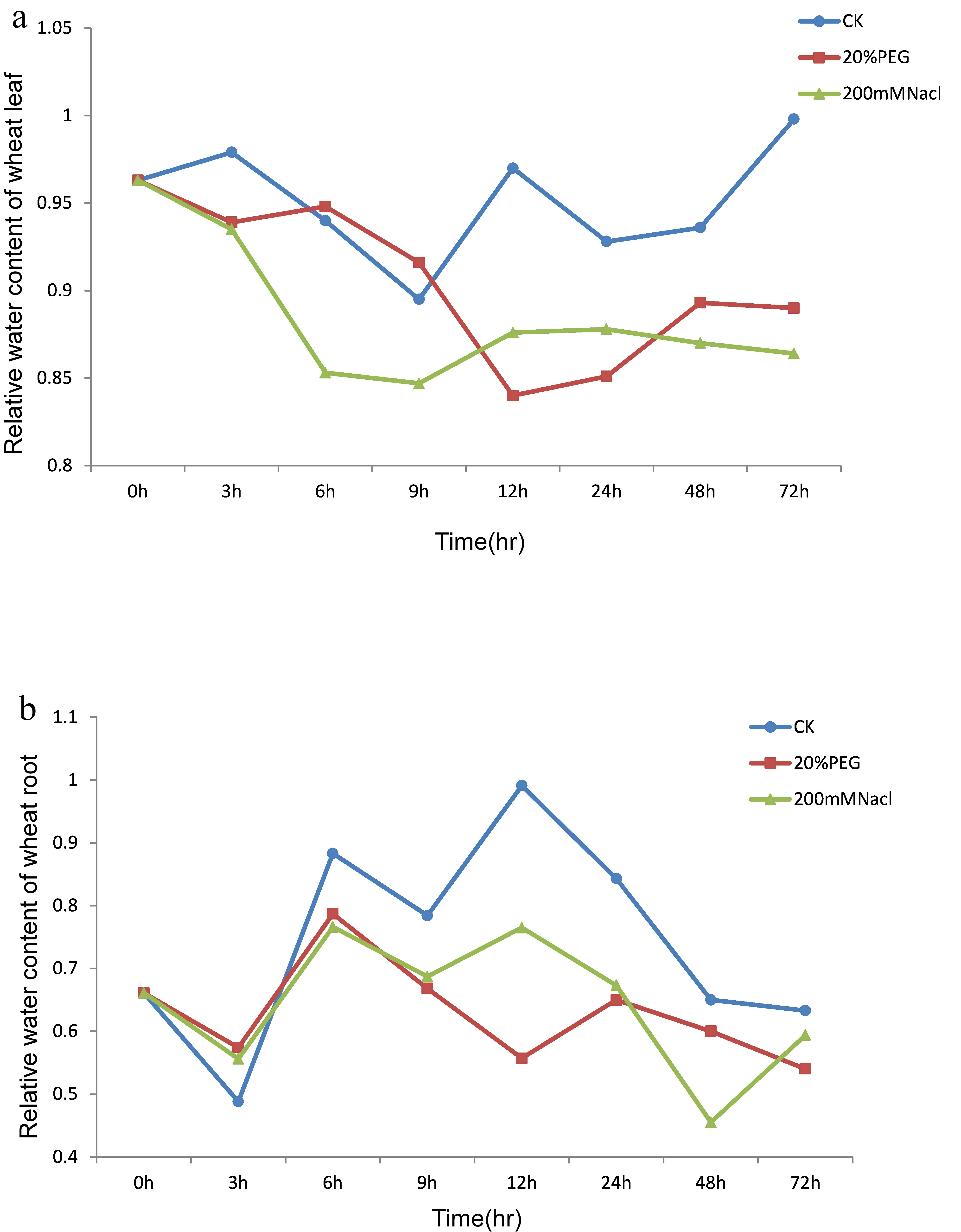

Supplement: Supplementary file 9 — Figure S4. Relative water content changes of wheat leaf and root under PEG and salt stress treatments. (JPG 1208 kb) [file 12864_2019_5455_MOESM9_ESM.jpg]

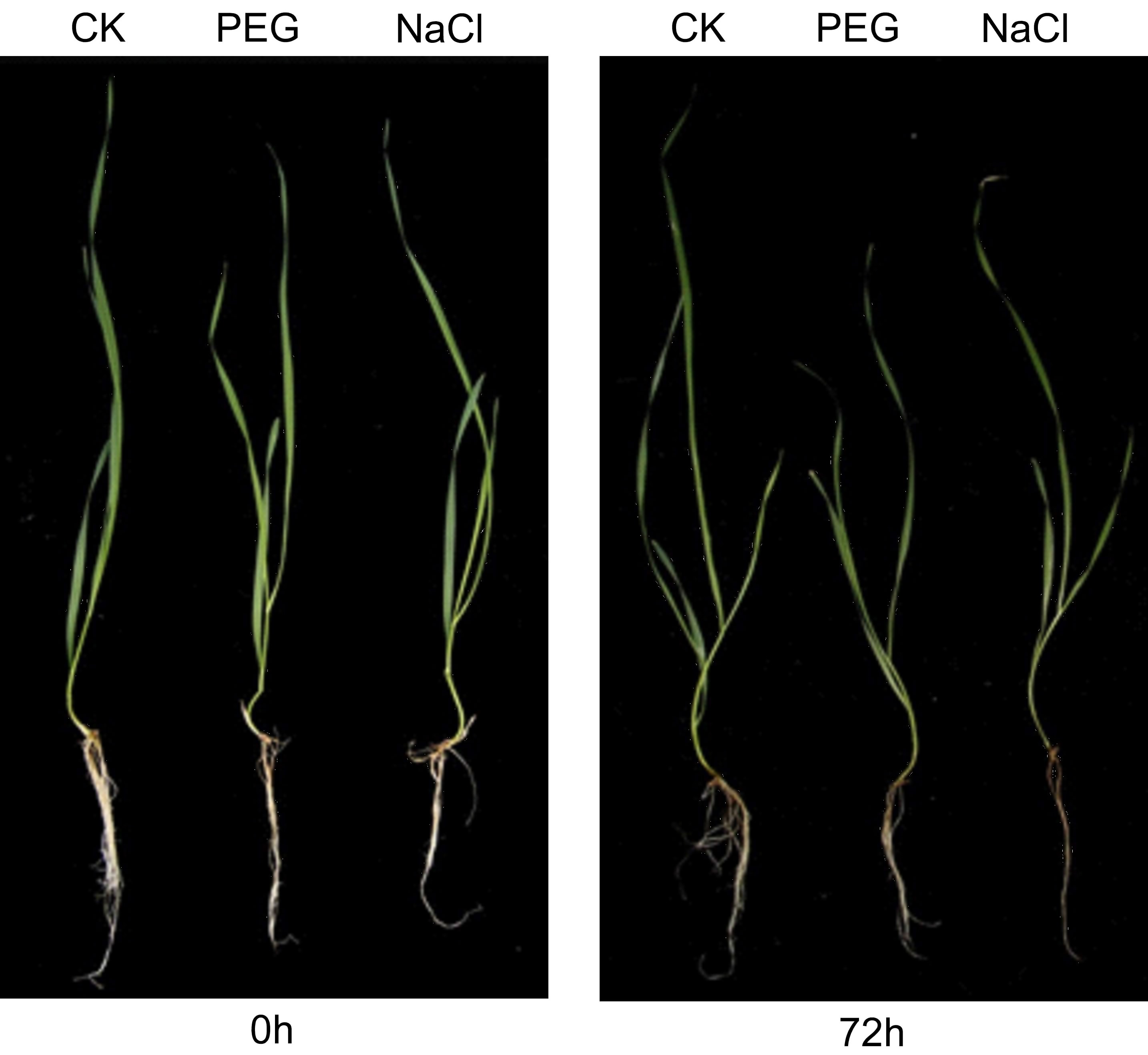

Supplement: Supplementary file 10 — Figure S5. The wheat seeding changes under 200 mM NaCl and 20% PEG6000. (JPG 236 kb) [file 12864_2019_5455_MOESM10_ESM.jpg]
